# Recurrent Pneumothoraces in a Patient With Anti-Sjögren’s Syndrome A Antibody-Positive Cystic Lung Disease: An Atypical Presentation of Sjögren’s Disease

**DOI:** 10.7759/cureus.81722

**Published:** 2025-04-04

**Authors:** Timothy N Holbrook, Lavanya Srinivasan, Stephen E Baker

**Affiliations:** 1 Internal Medicine, Baylor Scott & White All Saints Medical Center, Fort Worth, USA; 2 Pulmonology and Critical Care, Baylor Scott & White All Saints Medical Center, Fort Worth, USA; 3 Pathology, Baylor Scott & White All Saints Medical Center, Fort Worth, USA

**Keywords:** anti-ssa antibody, anti-ssb antibody, bronchiolitis, cystic lung disease, lymphocytic interstitial pneumonia, pneumothorax, pulmonary sjögren’s disease, secondary pneumothorax, sjögren’s disease

## Abstract

Sjögren’s disease (SD) is a systemic autoimmune disorder primarily characterized by sicca symptoms. However, pulmonary manifestations can occur and may precede glandular involvement. Spontaneous pneumothorax as an initial presentation of SD is exceedingly rare, with no prior reports in male patients. We report a case of a 54-year-old male who presented with recurrent, spontaneous pneumothoraces. Imaging revealed cystic lung disease, and histopathology demonstrated features of lymphoid interstitial pneumonia and constrictive bronchiolitis. A comprehensive autoimmune workup was notable for positive SSA-52 (Ro52) antibodies, leading to a diagnosis of pulmonary SD despite the absence of sicca symptoms. Following bilateral robotic-assisted bleb resection and pleurodesis, the patient recovered and was scheduled for continued pulmonary and rheumatologic monitoring. This case highlights the potential for SD to manifest as lung-predominant disease without classic sicca symptoms, which may result in diagnostic delays. The presence of recurrent pneumothorax and cystic lung disease in an otherwise healthy patient should prompt consideration of autoimmune etiologies, including SD.

## Introduction

Sjögren’s disease (SD) is a common autoimmune disease characterized by its most prevalent symptoms, xerostomia and xerophthalmia, or sicca syndrome [1]. Numerous extraglandular features can present in SD, including pulmonary manifestations such as xerotrachea, bronchiolitis, and interstitial lung disease (ILD) [2]. However, SD presenting with a primary manifestation of spontaneous pneumothorax has rarely been described in the literature. To our knowledge, no reports exist of this presentation in a male. 

We report a case of recurrent pneumothorax and cystic lung disease as an initial manifestation of primary SD without sicca symptoms. We also call for broader diagnostic criteria or alternative classification strategies for non-sicca, lung-predominant SD. 

## Case presentation

**Figure 1:**
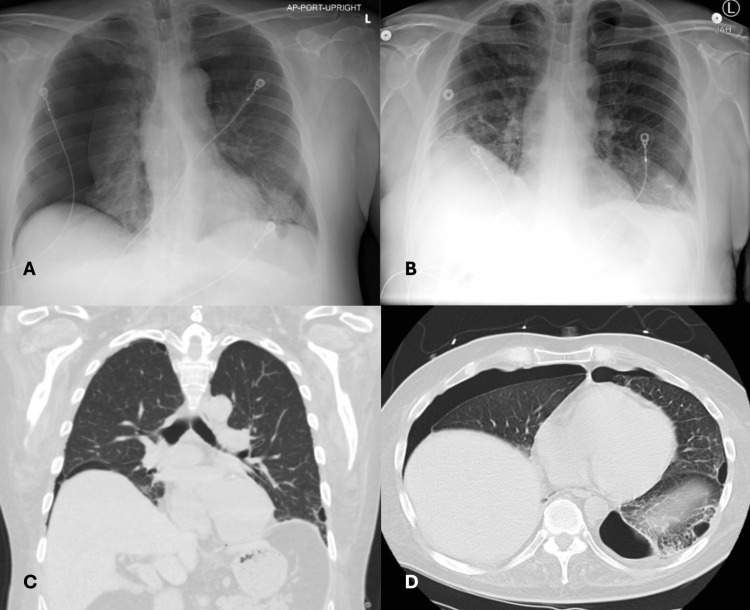
Initial and Subsequent Radiological Findings A) Chest radiography from initial presentation with bilateral pneumothoraces. B) Chest radiography from subsequent presentation with left apical pneumothorax. C) Computed tomography of the chest with lung cysts primarily at the bases. A few cysts are located at the apex. D) Sagittal plane view with bilateral pneumothoraces and lung bullae present at the left lung base.

A 54-year-old Caucasian male, a nonsmoker with recent bilateral pneumothoraces, presented with a recurrent pneumothorax discovered during preoperative evaluation. One month prior, he presented to our hospital with the complaint of worsening dyspnea on exertion and a nonproductive cough. He denied any recent trauma or significant smoking history. As a military veteran, he had been deployed to the Middle East but denied exposure to burn pits. His family history was unremarkable. Chest radiography at that time showed bilateral pneumothoraces (Figure 1A). Bilateral chest tubes were placed, and chest computed tomography (CT) revealed cystic lung disease primarily at the bases (Figure 1C). A robotic-assisted bleb resection, along with combined mechanical and chemical pleurodesis, was performed on the right thorax with plans for future intervention on the left side. 

**Figure 2:**
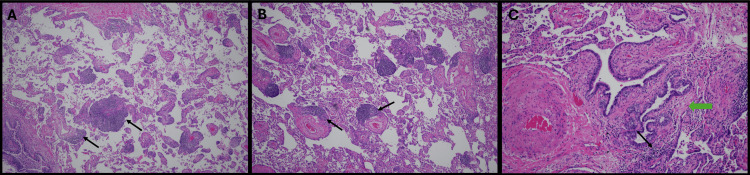
Microscopic Examination of Lung Biopsy A) Photomicrograph of the resected lung tissue demonstrating interstitial lymphocytic infiltrates (black arrows) expanding the alveolar septa (H&E, 4×). B) Numerous perivascular and interstitial lymphocytic infiltrates expanding the alveolar septa (H&E, 4×). C) Interstitial lymphocytic infiltrates and features of constrictive bronchiolitis, including fibrosis of the bronchial wall (green arrow) and bronchiolar metaplasia of adjacent alveolar septa (H&E, 20×) [3].

One month later, on preoperative evaluation, a recurrent pneumothorax on the left side was identified on chest radiography (Figure 1B), prompting his presentation to us. Upon admission for chest thoracostomy, his vitals were notable for tachycardia and an oxygen saturation of 98% on 2 liters via nasal cannula. Physical examination revealed normal oral mucosa with good dentition, no lymphadenopathy, skin lesions, or joint deformity. Robotic-assisted bleb resection and combined mechanical and chemical pleurodesis were performed on the left. Histopathological examination of the resected lung revealed emphysematous blebs in a background of features suggestive of constrictive bronchiolitis, including bronchioles with fibrotic walls and prominent bronchiolar metaplasia of the adjacent alveolar septa (Figure 2C). Prominent inflammatory infiltrates with numerous lymphoid aggregates were noted in the lung parenchyma, correlating with lymphoid interstitial pneumonia (LIP) (Figure 2A, 2B). Pleural tissue demonstrated fibroadipose tissue with fibrinous pleuritis. These specimens were similar to the samples examined after the initial resection and pleurodesis one month ago. Gram stain and cultures for acid-fast bacilli and fungus were negative. 

Additional diagnostic tests were conducted to identify the underlying etiology of the cystic lung disease. Complete blood counts, renal and liver function tests, and metabolic panels were within normal limits. Previously, alpha-1 antitrypsin was 151 mg/dL, within the normal range, and with an MM phenotype. C-reactive protein was 1.8 mg/dL and erythrocyte sedimentation rate was 20 mm/h. A comprehensive autoimmune workup was notable for positive SSA-52 (Ro52) antibody and cyclic citrullinated peptide (CCP) antibody (see Table 1 for full list of values). Based on serologic, histologic, and radiologic findings, the patient was diagnosed with recurrent spontaneous pneumothoraces secondary to pulmonary SD despite a lack of xerophthalmia or xerostomia. 

**Table 1:** Autoimmune Serologic Markers A1AT: alpha-1 antitrypsin; anti-SSA-52: Sjögren anti-SS-A-52 antibody; anti-SSA-60: Sjögren anti-SS-A-60 antibody; anti-SSB: Sjögren anti-SS-B antibody; ANA: antinuclear antibodies; RF: rheumatoid factor; anti-CCP: anti-cyclic citrullinated peptide antibody; anti-Scl-70: anti-scleroderma-70 antibody; anti-U3 RNP: anti-fibrillarin antibody; anti-Sm/RNP: anti-Smith/ribonucleoprotein particle antibody; anti-PL-12: antisynthetase antibody; anti-PL-7: antisynthetase antibody; anti-EJ: anti-glycyl-tRNA synthetase antibody; anti-OJ: anti-isoleucyl-tRNA synthetase antibody; anti-SRP: anti-signal recognition particle antibody; anti-PM/SCL-100: connective tissue disease anti-PM/SCL-100 antibody; anti-Mi-2: myositis anti-Mi-2 antibody; anti-P155/140: myositis anti-P155/140 antibody; anti-SAE1: dermatomyositis anti-SAE1 antibody; anti-MDA5: dermatomyositis anti-MDA5 antibody; anti-NXP2: myositis anti-NXP2 antibody; ANCA: antineutrophil cytoplasmic antibodies; ACE: angiotensin-converting enzyme.

Test	Result	Normal Range
A1AT	151 mg/dL	83-199 mg/dL
A1AT Phenotype	MM	
Anti-SSA-52 (Ro52)	157 AU/mL	0-40 AU/mL
Anti-SSA-60 (Ro60)	0 AU/mL	0-40 AU/mL
Anti-SSB (La)	1 AU/mL	0-40 AU/mL
ANA	Negative up to 1:160	
RF	13 IU/mL	Negative <14 IU/mL
Anti-CCP	>250 Units	Negative <20 Units
Anti-Scl-70	Negative	
Anti-U3 RNP	Negative	
Anti-Sm/RNP	Negative	
Anti-PL-12	Negative	
Anti-PL-7	Negative	
Anti-EJ	Negative	
Anti-OJ	Negative	
Anti-SRP	Negative	
Anti-Ku	Negative	
Anti-PM/SCL-100	Negative	
Anti-Mi-2	Negative	
Anti-P155/140	Negative	
Anti-SAE1	Negative	
Anti-MDA5	Negative	
Anti-NXP2	Negative	
ANCA	Negative at 1:20	
ACE	16 U/L	9-67 U/L
Aldolase	6.5 U/L	1.2-7.6 U/L

Postoperatively, the patient recovered and met the criteria for discharge following a brief period of conservative management. Ongoing pulmonary and rheumatologic surveillance is planned, including pulmonary function testing and high-resolution computed tomography once post-pleurodesis inflammation has sufficiently resolved. Ocular and salivary diagnostic testing have been deferred at this time due to the absence of clinical symptoms; however, the patient will be monitored for symptom development, and testing will be performed if indicated. 

## Discussion

While glandular involvement is by far the most frequent manifestation of SD, numerous extraglandular symptoms such as arthritis (76% of patients), cutaneous vasculitis (64% of patients), glomerulonephritis (28% of patients), and small-fiber neuropathy (80% of patients) have been encountered at diagnosis [4,5]. Pulmonary involvement occurs in 22% of primary SD patients and the presence of anti-SSA antibody is a predisposing risk factor [2,6]. Manifestations include xerotrachea, bronchiolitis, bronchiectasis, asthma, ILD, cystic lung disease, and lung-associated lymphomas [2]. These can vary in severity and presentation, explaining the wide variability among patients with SD. 

Cystic lung disease is an uncommon clinical and radiographic presentation but is found more commonly in SD compared to other connective tissue diseases [7]. The cystic pattern linked to SD typically presents, as seen in our patient, with a wide range of cyst sizes, internal structures within the cysts, perivascular and often basilar-predominant distribution, and frequent associations with ground-glass opacities and nodules [7]. Cyst formation has also been attributed to LIP, characterized by diffuse proliferation of polyclonal lymphocytes and plasma cells in the pulmonary parenchyma (Figure 1D) [2,8]. While LIP exists on a continuum with follicular bronchiolitis, other bronchiolitis types have been found in SD, including chronic bronchiolitis and constrictive bronchiolitis [2]. 

In our patient, pneumothorax likely developed as a result of the inherent structural weakness in the lung parenchyma from cyst formation combined with pleural weakening as evident by the fibrous tissue obtained on pleural biopsy. To the best of our knowledge, only three cases of pneumothorax as a primary manifestation of SD have been described [9-11]. All reported cases were females and had related sicca syndrome, in contrast to our patient. When lung involvement appears before the onset of sicca symptoms, an occult form of SD may be present, offering a diagnostic challenge [12]. 

The diagnostic criteria and classification systems for primary SD have evolved through multiple iterations, each demonstrating varying diagnostic performance [1]. Demonstration of autoimmunity via detection of serum antibodies, anti-SSA (Ro) and anti-SSB (La), or focal lymphocytic sialoadenitis on salivary gland biopsy is the most common confirmatory finding [1]. Anti-SSA-52 (Ro52) antibodies are less specific than anti-SSA-60 (Ro60) antibodies [13]. Conversely, anti-Ro52 is often associated with an increased risk of extraglandular manifestations in SD, including ILD [14]. Despite anti-Ro60 being more closely linked to SD itself, in the appropriate clinical context, anti-Ro52 should not be disregarded. 

However, in patients presenting with extraglandular manifestations consistent with SD, the absence of sicca symptoms can lead to premature dismissal and delay of diagnosis up to two years [15]. We support the EULAR-SS task force clinical recommendation that salivary gland biopsy and dry eye tests offer diagnostic confirmation but are not necessary to make the diagnosis of SD in the presence of a comprehensive diagnostic approach [5]. 

Interestingly, our patient had elevated anti-CCP antibodies detected in his serum. These antibodies are directed against peptides that contain citrulline and are highly specific for rheumatoid arthritis (RA) [16]. We consider this finding to be related to the timing of measurement, a few hours after mechanical and chemical pleurodesis. Recent surgery and trauma can lead to elevated levels of anti-CCP antibodies secondary to inflammation and resulting circulation of citrullinated proteins [17]. Additionally, cystic lung disease and findings of LIP are not common features of RA-associated ILD [18]. 

## Conclusions

While xerostomia and xerophthalmia are features of SD, extraglandular manifestations, such as cystic and ILD, can occur as the primary manifestation of SD. This case highlights recurrent spontaneous pneumothorax as a rare presentation of SD, emphasizing the need for increased clinical awareness in patients presenting with unexplained cystic lung disease and SSA positivity. Given that current classification criteria may overlook or delay diagnosis in non-sicca presentations, we advocate for a broader diagnostic approach that considers pulmonary manifestations as a potential primary feature of SD. Early recognition and multidisciplinary management of SD are essential for mitigating disease progression and optimizing patient outcomes. 
